# Validity and reliability of the Brazilian version of the reading anxiety scale (RAS) for children

**DOI:** 10.1590/1980-5764-DN-2024-0212

**Published:** 2025-10-27

**Authors:** Francielle Machado Beria, Giulia Moreira Paiva, Luciane da Rosa Piccolo, Vitor Geraldi Haase, Gisele Gus Manfro, Jerusa Fumagalli de Salles

**Affiliations:** 1 Atitus Educação, Porto Alegre RS, Brazil.; 2 Universidade Federal de Minas Gerais, Belo Horizonte MG, Brazil.; 3 NYU Grossman School of Medicine, NYU Langone Health NY, USA.; 4 Universidade Federal do Rio Grande do Sul, Porto Alegre RS, Brazil.

**Keywords:** Anxiety, Reading, Neuropsychology, Psychometrics, Child, Ansiedade, Leitura, Neuropsicologia, Psicometria, Criança

## Abstract

Reading anxiety (RA) significantly influences children’s emotional and cognitive development, affecting their reading performance. Effective identification of RA requires reliable and valid assessment tools tailored for specific populations, such as Brazilian children.

**Objective:**

To evaluate the psychometric properties, including validity and reliability, of the Brazilian version of the Reading Anxiety Scale (RAS) in elementary school children.

**Methods:**

A sample of 225 Brazilian elementary students was assessed using the RAS-20. Confirmatory factor analysis (CFA) was employed to evaluate content-based validity. Internal consistency was measured using Cronbach’s alpha, and Pearson’s correlations assessed associations with external constructs such as reading performance and executive functions. Group comparisons were conducted using ANCOVA between children with and without reading difficulties.

**Results:**

After CFA adjustments, a single-factor model with 18 items showed robust internal consistency. The correlation between RAS-20 scores and variables related to reading and cognitive functions supported moderate validity. Children with reading difficulties scored significantly higher on the RA scale than those without.

**Conclusion:**

The adapted 18-item RAS version is a reliable and valid tool for assessing reading anxiety in Brazilian children, contributing to more effective identification and intervention in educational contexts.

## RESUMO.

A ansiedade de leitura (AL) influencia significativamente o desenvolvimento emocional e cognitivo das crianças, afetando seu desempenho na leitura. A identificação eficaz da AL requer instrumentos de avaliação confiáveis e válidos, adaptados a populações específicas, como crianças brasileiras.

**Objetivo:**

Investigar as propriedades psicométricas, incluindo validade e confiabilidade, da versão brasileira da Escala de Ansiedade de Leitura (reading anxiety scale – RAS) em crianças do ensino fundamental.

**Métodos:**

Uma amostra de 225 estudantes brasileiros do ensino fundamental foi avaliada utilizando a RAS-20. A validade de conteúdo foi analisada por meio de análise fatorial confirmatória (AFC). A consistência interna foi verificada pelo alfa de Cronbach, e as correlações de Pearson foram utilizadas para avaliar associações com construtos externos, como desempenho em leitura e funções executivas. Comparações entre grupos foram realizadas por meio de ANCOVA entre crianças com e sem dificuldades de leitura.

**Resultados:**

Após ajustes na AFC, um modelo unifatorial com 18 itens apresentou forte consistência interna. As correlações entre os escores da RAS-20 e variáveis cognitivas e de desempenho de leitura indicaram validade moderada. Crianças com dificuldades de leitura apresentaram escores significativamente mais altos na escala de AL em comparação àquelas que sem dificuldades.

**Conclusão:**

A versão brasileira adaptada de 18 itens da RAS é um instrumento válido e confiável para avaliar a ansiedade de leitura em crianças brasileiras, contribuindo para uma identificação e intervenção mais eficazes em contextos educacionais.

## INTRODUCTION

 Understanding the various manifestations of anxiety is critical, given its high global prevalence, ranging from 0.9 to 28.3%^1^. Among the various forms of anxiety, reading anxiety (RA) is recognized as a distinct and multifaceted response involving cognitive, emotional, physical, and behavioral components^2,3^. Unlike general anxiety, RA arises specifically in reading contexts and can directly impair performance by disrupting decoding, fluency, and comprehension^4^. Over time, this may lead children to avoid reading activities altogether, further compounding learning difficulties. Recent studies show that working memory, phonological awareness, and processing speed are important for the development and persistence of reading anxiety^5,6^. These findings point to a complex interplay between emotional and cognitive systems in reading acquisition, reinforcing the need for specialized assessment tools to detect and address RA early on. 

 Although several instruments for assessing reading anxiety exist internationally, they vary in theoretical grounding and psychometric rigor^5,7^. Some are adapted from unrelated anxiety measures, while others lack validation in diverse populations. In Brazil, Piccolo et al.^7^ adapted the original Reading Anxiety Scale (RAS) by Zbornik and Wallbrown^3^ into a 20-item short version, providing preliminary evidence of reliability and convergent validity with general and math anxiety. However, their findings were based on a small, homogeneous sample, and further psychometric analysis — particularly factor structure and generalizability — is needed. 

 Given Brazil’s cultural and educational diversity, a validated tool tailored to the Brazilian context is essential for identifying students at risk for RA. Such tools can support targeted educational interventions and promote equitable literacy development. Therefore, the present study aimed to expand upon prior work by examining the validity and reliability of the Brazilian version of the RAS in a larger, more diverse sample of elementary school children. In doing so, it sought to provide more robust evidence for the scale’s applicability in research and educational settings across Brazil. 

 In the existing literature, diverse reading anxiety instruments with varied theoretical foundations have been utilized, including those adapted from sources such as math anxiety measures^5^ and foreign instruments^7^. Regardless of the instrument’s origin, further conceptualization, as well as rigorous assessment of validity and reliability, are imperative for effectively evaluating RA. 

 In international literature, the RAS by Zbornik and Wallbrown^3^ assesses RA in English L1 children aged 9 to 12. The Brazilian version, adapted by Piccolo et al.^7^, condensed the scale to 20 items. This adaptation demonstrated good reliability and convergent validity with general and math anxiety scales. Moreover, it showed validity through correlations with external variables, including decoding of isolated words and pseudowords, word reading fluency, and text reading fluency. Building on the preliminary evidence presented by Piccolo et al.^7^, the current study aimed to expand the investigation of the psychometric properties of the Brazilian RAS using a larger sample of elementary school children compared to the original study. 

## METHODS

### Participants

 Data were compiled from four distinct samples collected in two Brazilian states, Minas Gerais and Rio Grande do Sul. Employing convenience sampling, the study included 225 children aged 7 to 11 years, enrolled in second to fifth grades at both public and private elementary schools. Sociodemographic information for the entire sample and by state is presented in Table 1. 

**Table 1:** Sociodemographic characteristics of the total sample and by data collection state.

Characteristics		Total sample	RS (n=136)	MG (n=89)
		Mean (SD; range) or n (%)		
Age (years)		8.9 (0.1; 7–11)	8.7 (0.7; 8–10)*	9.2 (1.2; 7–11)*
Gender (female)		104 (46)	61 (45)	40 (48)
School year (grade)				
	2^nd^	27 (12)	14 (10)	10 (12)
	3^rd^	112 (50)	83 (62)*	28 (34)*
	4^th^	58 (26)	38 (28)*	18 (22)*
	5^th^	28 (12)	0*	27 (32)*
School type – public		176 (78)	87 (64)	89 (100)
School type – private		49 (22)	49 (36)*	0*
Ideb (n=121)		7.0 (0.5; 6–7.5)	6.5 (0.5; 6–7)	7.3 (0.2; 6.9–7.5)
ABEP		30.8 (9.4; 7–67)	31.8 (11.5; 7–67)	30.1 (7.1; 18–45)
	A	10 (10)	8 (16)*	2 (4)*
	B1	11 (11)	5 (10)	6 (12)
	B2	36 (35)	15 (29)	21 (41)
	C1	28 (27)	15 (29)	13 (25)
	C2	15 (15)	6 (12)	9 (18)
	D–E	2 (2)	2 (4)	0
RAS		68.0 (11.9; 28–90)	69.1 (11.6; 40–89)	66.3 (12.3; 28–90)

Abbreviations: SD, standard deviation; RS, Rio Grande do Sul; MG, Minas Gerais; Ideb, Índice de Desenvolvimento da Educação Básica; ABEP, Associação Brasileira de Estudos Populacionais; RAS, Reading Anxiety Scale (20 items).

Note: *p<0.05.

### General procedures and ethical considerations

 The studies were approved by the Research Ethics Committees at Universidade Federal de Minas Gerais (UFMG) and Universidade Federal do Rio Grande do Sul (UFRGS) under protocol numbers 939.562. 1.023.371, and 1.632.099/2016. Upon school approval, Informed Consent forms and questionnaires assessing family socioeconomic status^8^ and children’s health, as well as the Multimodal Treatment Study for Attention-Deficit/Hyperactivity Disorder Swanson, Nolan, and Pelham, Version IV (MTA-SNAP-IV scale^9^), were sent to parents. 

 Subsequently, the research instruments were administered in both group and individual settings. All assessments were conducted in a randomized order by trained researchers with experience in developmental neuropsychology/psychology. Upon completion of data collection, comprehensive performance reports for each child were provided to participating caregivers and schools. 

### Measures

 The selected instruments covered cognitive, academic, and psychological domains known to be associated with reading anxiety. All tests used were validated for the Brazilian population and provided a comprehensive profile of the participants. 

#### Exclusion criteria

- Raven’s Colored Progressive Matrices Test^10^
- Health and education questionnaire.
- MTA-SNAP-IV^9^

#### Sociodemographic variables

- Brazilian Economic Classification Criteria^8^
- Basic Education Development Index (*Índice de Desenvolvimento da Educação Básica* – Ideb)^11^

#### Reading anxiety

- Reading Anxiety Scale (RAS-20)^7^

#### Reading and phonological awareness

- Word and Pseudoword Reading Task (WPRT)^12^
- Textual Reading Fluency (TRF)^13^
- Word Reading Fluency Test (WRFT)^14^
- Phoneme suppression task^15^

#### Numerical cognition skills

- Numerical Transcoding Task (NTT)^16^

#### Writing

- Word and pseudoword spelling task (WPST)^17^

#### Working memory

- Digit span^18^
- Letter span^19^
- Corsi-Tapping Blocks^20^

#### Executive functions

- Contingency Naming Task^19,21^
- Verbal Fluency^22^
- Five Digit Test^23^

#### Academic performance

- Test of School Performance^24,25^
- Psychological variables
- MTA-SNAP-IV^10^
- Reading Motivation Scale^26^
- Math Anxiety Questionnaire^27^

### Data analysis

 Descriptive analyses, including frequencies, means, and standard deviations, were used to describe the sample’s sociodemographic and school characteristics (age, gender, education, type of school, Ideb, and socioeconomic level), as well as scores on the RAS and other outcome measures. State comparisons (Rio Grande do Sul – RS and Minas Gerais – MG) utilized *t*-tests for continuous variables (RAS-20, age, Ideb, and ABEP [socioeconomic status index]) and chi-square tests for categorical variables (gender, grade, type of school, and socioeconomic classification according to ABEP). 

 In the content-based validity study of the RAS, confirmatory factor analyses (CFA) from Piccolo et al.^7^ were replicated with a larger and more diverse sample (N=225 *vs.* N=88). CFA aimed to examine the three-factor structure of the Brazilian version proposed by Piccolo et al.^7^. Models were assessed using Kaiser-Meyer-Olkin (KMO) index (threshold 0.60), Bartlett’s test of sphericity (p<0.05), factors with eigenvalues greater than 1, and items with loadings >0.40, following the criteria of Hair et al.^28^. Additionally, overall model fit was examined using multiple indices, including the ꭓ^2^ test, Comparative Fit Index (CFI), Tucker-Lewis Index (TLI), Root Mean Square Error of Approximation (RMSEA), and Standardized Root Mean Square Residual (SRMR). These indices were used to assess how well the proposed factor structure captured the data, following conventional guidelines for model fit interpretation. The Scree test method^29,30^ was used for graphical analysis of factor dispersion until a horizontal or sharp drop in the curve was reached. To enhance accuracy, parallel analysis (PA)^31^ was also utilized, as it is less influenced by sample size and item loadings^32^. PA involves comparing real data eigenvalues with those from a hypothetical matrix generated through Monte-Carlo simulation. 

 In terms of reliability, internal consistency was assessed using Pearson’s correlations and the alpha coefficient for the RAS items. Validity was established through Pearson’s correlation with external variables, and group comparisons were performed using Analysis of Covariance (ANCOVA) to assess RAS performance between groups with and without reading difficulties. Reading difficulty was defined by the 7^th^ percentile cutoff on WPRT^12^, with grade, gender, and type of school as covariates. Due to varying participant assessments, the sample size for correlation analysis ranged from 46 to 225. All analyses were conducted using SPSS 26 software, with a significance level set at 5%. 

## RESULTS

### Descriptives

Table 1 shows sociodemographic characteristics and RAS scores. Significant differences were observed between the samples in age, school years (third to fifth grades), school type (private), and ABEP-SES (Class A). No significant differences were found in mean RAS scores between states (RS and MG), allowing for combined analysis of RAS data from both states. 

### Factor analysis of the reading anxiety scale

 The factor analysis of the RAS, using principal axis factoring (PAF) and Promax rotation, demonstrated good fit indices (KMO=0.83, Bartlett’s test χ^2^ (190)=1,089.82, p<0.001). However, the total explained variance of 38.78% across three factors fell below the acceptable threshold of 0.60, indicating that the factor structure did not account for a substantial portion of the variability in the data. This limitation suggests that the three-factor model may not fully capture the complexity of reading anxiety as measured by the RAS. 

 Additionally, the scree plot analysis supported a three-component model, but items shifted between Independence and Difficulty dimensions, likely due to strong correlations (r=0.75, p<0.001). Items 14 and 18 showed low correlation (r=0.35, p<0.001) and caused semantic confusion, rendering them inadequate for assessing reading anxiety. 

 Given the limited sample size in the study by Piccolo et al.^7^, the 20-item model was reevaluated, excluding items 14 and 18 based on the present factor analysis and a literature review^33^. However, these items continued to load on multiple factors, as shown in Table 2. Traditional methods, such as eigenvalue and Scree plot analyses, are limited in capturing complex structures^32^, which prompted the use of PA with 500 permutations. PA indicated a one-factor solution as the most suitable, further supporting the decision to adopt a one-factor structure in this study, as detailed in Table 2. 

**Table 2:** Parallel analysis to determine the number of factors to be extracted.

Number of factors	% of variance explained by actual data	% of variance explained by random data
1	38.78	12.92
2	9.99	11.39
3	6.85	10.5

Note: According to the parallel analysis, only one factor should be extracted, considering that, from the second factor, the variance explained by the random data becomes greater than that explained by the real data.

 The overall model fit indices showed a mixed pattern. Incremental fit indices suggested an acceptable fit (CFI=0.930; TLI=0.920), whereas residual-based indices exceeded recommended thresholds (RMSEA=0.335, 90%CI [0.325–0.346]; SRMR=0.301). The ꭓ^2^ test was significant, χ^2^ (190)=3,051.53, p<0.001, which is common in large models. 

Table 3 illustrates the item allocations and factor loadings resulting from the CFA, showing the original factor assignments from Piccolo et al.’s^7^ study alongside the factor loadings identified in the present analysis. 

**Table 3:** Factor analysis of the reading anxiety scale (n=225).

Components*	Items (English)	Items (Brazilian Portuguese)	Factors		
			1	2	3
d	I can read, but I can’t understand what I have read.	4. Eu consigo ler, mas eu não consigo entender o que eu leio.	0.64	0.36	0.36
in	I cannot improve my reading without help.	11. Eu não consigo melhorar minha leitura sem ajuda.	0.59	0.34	0.53
in	I like someone to help me when I do my reading homework.	16. Eu gosto que alguém me ajude quando estou fazendo os deveres de casa de leitura.	0.58		
in	I need help in reading.	3. Eu preciso de ajuda para ler.	0.58		0.38
d	I quickly forget what I have read, even if I have just read it.	9. Eu esqueço rapidamente o que eu li mesmo que eu tenha acabado de ler.	0.55	0.39	0.33
in	The teacher helps me when we are reading.	5. O(a) professor(a) me ajuda quando estamos lendo.	0.54		
d	Reading is hard on my eyes.	1. Na minha opinião, ler é difícil	0.53	0.30	0.35
d	I have a difficult time understanding what I have read.	2. Tenho dificuldade em compreender o que leio.	0.50		0.31
in	If no one helped me, I would fail reading.	8. Se ninguém me ajudasse, eu não conseguiria ler.	0.42		0.37
e	I do not like to read.	12. Eu não gosto de ler.	0.30	0.83	
e	I can read, but I don’t feel like it.	10. Eu consigo ler, mas eu não gosto.	0.37	0.70	0.38
e	I become upset when there is a lot to read.	20. Eu fico chateado quando tem muita coisa para ler.	0.45	0.60	
e	I don’t like to look at books.	15. Eu não gosto de olhar para livros.	0.30	0.57	
e	I don’t like to look at books	19. Olhar para os livros me deixa chateado e/ou nervoso.		0.54	
in	No matter how hard I try, I just can’t read well.	6. Não importa o quanto eu tente, eu não consigo ler bem.	0.47		0.68
in	I cannot read well without the help of others.	13. Eu não consigo ler bem sem ajuda de outras pessoas.	0.60	0.29	0.66
d	I am not smart enough to read well.	17. Eu não sou inteligente o suficiente para ler bem.		0.33	0.49
d	People who read well are not like me.	7. Pessoas que leem bem não são como eu.	0.41	0.33	0.46

Note: *Components as proposed by Piccolo et al.^7^: "d": difficulty (perception of reading as a challenging task), "in": independence (confidence in independent reading) and "e": enjoyment (interest in reading). Original factor loadings and items Portuguese translation were published in Piccolo et al.^7^.

### Reliability analysis

Table 4 presents statistically significant correlations between the scale items, which ranged from 0.20 to 0.65. In addition, the scale demonstrated adequate internal consistency, with a high Cronbach’s alpha of 0.87 for the entire scale^34^. 

**Table 4:** Item correlations.

	1	2	3	5	6	7	8	9	10	11	12	13	14	16	17	18	20	21
1	-																	
2	0.36*	-																
3	0.38*	0.27*	-															
5	0.44*	0.37*	0.38*	-														
6	0.20*	0.30*	0.35*	0.33*	-													
7	0.25*	0.25*	0.31*	0.35*	0.25*	-												
8	0.18†	0.17†	0.13	0.31*	0.28*	0.35*	-											
9	0.20*	0.17†	0.36*	0.19†	0.16†	0.26*	0.33*	-										
10	0.30*	0.26*	0.24*	0.50*	0.27*	0.34*	0.32*	0.23*	-									
11	0.23*	0.19*	0.16†	0.25*	0.04	0.26*	0.31*	0.22*	0.36*	-								
12	0.38*	0.28*	0.33*	0.37*	0.31*	0.43*	0.28*	0.28*	0.28*	0.34*	-							
13	0.24*	0.22*	0.10*	0.22*	0.12	0.18†	0.30*	0.08	0.24*	0.65*	0.29*	-						
14	0.35*	0.32*	0.46*	0.31*	0.33*	0.45*	0.36*	0.33*	0.25*	0.37*	0.60*	0.22*	-					
16	0.24*	0.22*	0.18†	0.27*	0.16†	0.15†	0.18†	0.09	0.14	0.34*	0.22*	0.48*	0.24*	-				
17	0.26*	0.25*	0.30*	0.33*	0.39*	0.23*	0.29*	0.29*	0.26*	0.13	0.37*	0.09	0.34*	0.21*	-			
18	0.19††	0.26*	0.11	0.24*	0.24*	0.40*	0.27*	0.10	0.21*	0.25*	0.21*	0.25*	0.28*	0.35*	0.14	-		
20	0.20*	0.10	0.08	0.20*	0.04	0.20*	0.16†	0.06	0.18†	0.32*	00.13	0.41*	0.16†	0.48*	0.15†	0.28*	-	
21	0.17†	0.22*	0.16†	0.28*	0.26*	0.13	0.16†	0.12	0.32*	0.42*	0.26*	0.46*	0.17†	0.27*	0.32*	0.05	0.31*	-

Note: *p<0.05; †p<0.01.

### Validity evidence based on correlations with instruments evaluating related constructs

 Only statistically significant results are presented in Table 5. RAS was significantly correlated with performance in written language, phoneme suppression (phonological awareness), working memory, executive functions, school performance (writing and arithmetic), and psychological variables. Notably, RAS correlated with time-dependent measures of TRF and phonological awareness (phoneme suppression task), as well as the spelling task of the school performance test (*teste de desempenho escolar* – TDE). 

**Table 5:** Correlations with other variables.

Domains	Scales		RAS-18
Reading	WPRT		
		Total score (n=183)	0.27*
		Score – regular words (n=183)	0.33*
		Score – irregular words (n=183)	0.26*
	TRF		
		Time in minutes (n=51)	-0.33†
		Words correctly read per minute (n=51)	0.37*
	Phonological awareness – Phoneme suppression task		
		Total score (n=87)	0.29*
	WRFT		
		Total score (n=184)	0.30*
Writing	WPST		
		Total score (n=73)	0.42*
		Words writing (n=73)	0.39*
		Pseudoword writing (n=86)	0.32*
	NTT Total Score (n=88)		0.22†
	TDE		
		Writing total score (n=88)	0.35†
Working memory	Digit span		
		Forward – Span (n=133)	0.18†
		Backward – Total numbers of trials (n=87)	0.23†
		Backward – Total numbers of correct trials (n=87)	0.24†
		Backward – Total of items (n=87)	0.23†
		Backward – Span (n=133)	0.17†
		Total score (span x correct trials) (n=133)	0.19†
	Visual working memory – Corsi		
		Forward – Total numbers of trials (n=87)	0.21†
		Backward – Total numbers of correct trials (n=87)	0.26†
		Backward – Total numbers of trials (n=87)	0.31*
		Backward – Span (n=87)	0.30*
		Total score (span x correct trials) (n=87)	0.23†
Executive functions	CNT		
		Inhibition – Reaction time (n=84)	-0.22†
		Letters – Reaction time (n=124)	-0.18†
		Numbers – Reaction time (n=84)	-0.22†
	Verbal fluency		
		Letter M – Total score (n=87)	0.40*
	FDT		
		Choose – Reaction time (n=86)	-0.31*
		Alternation – Reaction time (n=86)	-0.29*
		Inhibition – Reaction time (n=86)	-0.31*
		Flexibility – Reaction time (n=86)	-0.28*
Psychological variables	Reading motivation		
		EML (n=46)	0.44*
	MAQ		
		Total score (n=116)	-0.36*
		Scale A (n=116)	-0.49*
		Scale B (n=116)	-0.24*
		Scale C (n=116)	-0.29*

Abbreviations: RAS, reading anxiety scale; WPRT, Word/pseudoword Reading task; TRF, Textual reading fluency; WRFT, Word reading fluency test; WPST, Word and pseudoword spelling task; NTT, Numerical transcoding task; TDE, School performance test; CNT, Contingency Naming Task; FDT, Five Digit Test; EML, *Escala de Motivação para Leitura*; MAQ, Mathematics Anxiety Questionnaire; Scale A, self-perceived performance; Scale B, attitudes in mathematics; Scale C, unhappiness related to problems in mathematics.

Note: *p<0.01; †p<0.05.

 In working memory, significant correlations were observed with the Corsi backward and forward span tasks. Additionally, RAS was correlated with executive function variables, including reaction time on the five digit test (FDT) and Contingency Naming Task (CNT), as well as scores on the verbal fluency task. 

 Among the psychological variables, significant correlations were found with three of the four Math Anxiety Questionnaire (MAQ) subscales measuring different aspects of mathematics anxiety: "self-perceived performance" (Scale A), "attitudes in mathematics" (Scale B), and "unhappiness related to problems in mathematics" (Scale C). A significant correlation was also found with the Motivation for Reading Scale (MRS) subscale, which relates to demotivation for reading. 

### Validity evidence based on comparisons between groups

 RAS scores were compared between two groups: those with reading difficulty — defined as children scoring at or below the 7^th^ percentile on WPRT^12^ — and those without difficulty, scoring above the 7^th^ percentile. The groups were further stratified by grade, gender, and type of school. 

 Children with reading difficulties scored higher on the RAS than those without difficulties (M=68.23 vs. 62.04; F(1,174) = 4.79; p=0.03). The effect size of reading difficulty on reading anxiety was weak (partial η^2^=0.027). Adjustments for grade, gender, and school type were included in the RAS score analysis. 

## DISCUSSION

 This study aimed to examine the validity and reliability of the 20-item Brazilian version of RAS. The scale’s factor structure varied in item distribution from the previous version but sustained a comparable level of internal consistency with fewer items. Additional validity evidence, based on association with instruments measuring related constructs and reading performance criteria, was presented — complementing the study conducted by Piccolo et al.^7^ and supporting the validity of the scale for assessing reading anxiety in Brazilian children. 

 The factor analysis suggested a single-factor measure of reading anxiety, which is aligned with existing literature^4,6^, although it differs from the three-factor structure suggested in previous research with RAS^7^. Results from this study indicated good internal consistency and further validation analysis (discussed below) supports the scale’s adequacy for the Brazilian population. 

 The significant associations with other instruments assessing related constructs provide support for the validity of RAS and offer insights into the underlying mechanisms of reading anxiety. Regarding neuropsychological measures, RAS showed significant associations with tasks involving written language, phonological awareness, working memory, and executive functions, indicating that higher anxiety was associated with lower performance. These findings corroborate previous studies with the RAS^7^ and research on reading anxiety^6,35^. Interestingly, the significant correlations between reading anxiety and completion time for reading fluency, phonological awareness, spelling, and executive function tasks suggests an association with temporal demands in task execution. Based primarily on literature on general anxiety, it has been hypothesized that anxiety symptoms may overload critical cognitive resources such as attention and working memory^4,36^, thereby compromising performance in tasks that require heightened attention or short-term memory, such as reading aloud^37^. 

 Regarding correlations with psychological variables, significant associations were identified with three subscales of the math anxiety scale^27^: "Self-perceived performance" (Scale A), "Attitudes toward mathematics" (Scale B), and "Unhappiness related to math problems" (Scale C), corroborating previous findings^7^. No correlation was found with the MAQ subscale “Anxiety related to problems in mathematics” (Scale D), indicating the distinct nature of math anxiety and reading anxiety as constructs, despite shared associated factors indicated by correlations with specific MAQ subscales. Broad associations between reading anxiety and math anxiety have been reported in recent studies^35^. Furthermore, a correlation was observed with the demotivation subscale for reading from the Learning Motivation Scale (LMS). This finding aligns with previous studies^35^ and supports models by Katzir et al.^5^ and Ramirez et al.^4^ that highlight the complex relationship between motivation and the expression of reading anxiety. Further studies are needed to understand the role of intrinsic and extrinsic motivation in reading and its association with reading anxiety. For instance, an intrinsically motivated child may be more resilient and less susceptible to anxiety, as their primary focus may be on the enjoyment of reading, whereas a child driven by external factors — such as the need for approval or fear of negative consequences — may be more likely to experiencing anxiety regarding reading activities. 

 Validation analysis using a reading performance criterion indicated that children with reading difficulties showed higher levels of reading anxiety compared to those without reading challenges, even after accounting for gender, grade, and school type. This finding is consistent with previous research^38^ suggesting an association between the severity of reading difficulties and anxiety symptoms broadly, and reading anxiety specifically^35^. Notably, a bidirectional relationship between reading anxiety and performance has been hypothesized and supported by correlational evidence: children may avoid reading due to anxiety, thereby affecting their performance, while low reading performance may worsen anxiety^4^. This bidirectional association may be further compounded by environmental, cognitive, and emotional factors, such as those discussed above. 

 This study, while contributing valuable insights into the validity and reliability of RAS in the Brazilian context, is not without limitations. The assessments were conducted with a specific demographic of Brazilian children, which may raise concerns about the generalizability of the findings, as cultural, socioeconomic, and educational variations may impact the interpretation of reading anxiety. Additionally, the cross-sectional design may limit interpretation of potential causal relationships, highlighting the need for future research with longitudinal designs to explore the dynamics between reading anxiety, cognitive factors, and performance over time. Furthermore, the inherent subjectivity in self-report measures may result in bias. A limitation of the analysis is that some fit indices, particularly RMSEA and SRMR, were slightly above commonly recommended thresholds, suggesting that the model’s fit could be improved. Additionally, the model explained a relatively modest portion of the variance in the data, indicating that there may be additional factors contributing to reading anxiety that were not fully captured by the current model. These considerations point to the potential for refining the factor structure in future studies to better account for the observed variability. Recognizing these limitations, future research should address these aspects to provide a more comprehensive understanding of reading anxiety in diverse contexts. 

## Data Availability

The datasets generated and/or analyzed during the current study are available from the corresponding author upon reasonable request.
